# Weight-Based Bisphosphonate Administration for Multiple Myeloma Patients and the Risks of Skeletal Complications

**DOI:** 10.3390/jcm12041637

**Published:** 2023-02-18

**Authors:** Ji Hoon Bahk, Woo-Lam Jo, Soon-Yong Kwon, Hyung Chul Park, Young Wook Lim

**Affiliations:** Department of Orthopedic Surgery, Seoul St. Mary’s Hospital, School of Medicine, The Catholic University of Korea, 222, Banpodae-ro, Seocho-gu, Seoul 06591, Republic of Korea

**Keywords:** bisphosphonate, multiple myeloma, atypical femoral fracture, medication-related osteonecrosis of the jaw, safety limit

## Abstract

High-dose bisphosphonate for multiple myeloma patients might elevate risks of skeletal complications earlier than general expectations. This study aims to find incidences of atypical femoral fracture (AFF) and medication-related osteonecrosis of the jaw (MRONJ), elucidate their risk factors, and suggest cut-off values for the safer dosing of high-dose bisphosphonate treatment. By using the clinical data warehouse of a single institute, retrospective cohort data of multiple myeloma-diagnosed patients with high-dose bisphosphonate (pamidronate or zoledronate) treatment from 2009 to 2019 was extracted. Among 644 patients, the incidence of prominent AFF requiring surgery was 0.93% (6/644) and MRONJ was diagnosed in 11.8% (76/644) of the study population. For both AFF and MRONJ, the total potency-weighted sum of total dose per body weight (OR = 1.010, *p* = 0.005) were significant on logistic regression. Cutoffs of the potency-weighted total dose (mg) per body weight (kg) for AFF and MRONJ were 77.00 and 57.70 mg/kg, respectively. After roughly one year of treatment with high-dose zoledronate (about four years for pamidronate), an earlier thorough re-evaluation of skeletal complications should be taken. Body weight adjustments for accumulative dose calculation in terms of permissible dosing should be taken into consideration.

## 1. Introduction

Multiple myeloma (MM) is a type of hematologic malignancy caused by the accumulation of cancerous plasma cells in the bone marrow with substantial intra-clonal genetic heterogeneity. Neoplastic clones secrete immunoglobulins (Ig) or fragments of Ig, such as IgG, IgA, or light chains [1], which can be detected for a confirmed diagnosis of the disease [2]. Disturbed cell signaling exceedingly increases osteoclast activity and decreases osteoblast activity causing catastrophic results. MM is the most common primary malignancy of bone which frequently creates its characteristic multiple purely lytic punched-out lesions without reactive sclerosis [3,4]. Bone pain is a common complaint and structural vulnerability leads to pathologic fractures, which disable the patient.

Although the mainstay of the treatment of MM had been autologous hematopoietic stem cell transplantation in combination with induction and maintenance chemotherapies including steroids [5], administration of high-dose bisphosphonate has been proven to show considerable effects in preventing skeletal complications of MM. Protein prenylation is inhibited by the nitrogen-containing bisphosphonates (e.g., pamidronate and zoledronic acid), which interfere with mature osteoclasts and also its precursors [6,7]. 

Current hematologic guidelines of bisphosphonate administration on multiple myeloma patients to treat myeloma bone disease (MBD) mainly recommend intravenous pamidronate 90 mg every three to four weeks or intravenous zoledronate 4 mg every three to four weeks for up to 24 months [8,9,10]. Re-evaluation for the disease response after 24 months of treatment is usually recommended to consider discontinuation of the bisphosphonates at the physician’s discretion [8,9,10,11,12,13]. Considering that the typical treatment of osteoporosis is recommended to intravenously administer 30 mg every three months for pamidronate and 5 mg once a year for zoledronate [14,15], relative dosing for multiple myeloma is about 9.75 times and 10.40 times higher, respectively.

Prolonged higher dose treatment in a relatively concentrated period ironically increases the risks of skeletal complications from using bisphosphonates: atypical femur fractures (AFF) and medication-related osteonecrosis of the jaw (MRONJ) [16]. For the treatment for osteoporosis, incidences were reported to be 0.025–4.8% [17,18] and 0.3–6.7% [19] around the globe, respectively, and recent reports in South Korean cohorts were 0.13% (13/10,333) [20] and 0.10% (166/164,926), respectively for AFF and MRONJ [21]. Preventive measures for these potentially devastating complications also need attention.

Bisphosphonates and their clinical complications have been widely studied along with in vitro and in vivo studies regarding the efficacy and safety of nitrogen-containing bisphosphonates for the treatment of myeloma or bone metastases. Yet the literature focusing on skeletal complications of high-dose bisphosphonate treatment, especially in MM, are scarce. This study aims to elucidate long-term incidences of major skeletal complications and their risk factors and to further suggest cut-off values for safe dosing when prescribing high-dose bisphosphonates in multiple myeloma patients. 

## 2. Materials and Methods

**Figure 1:**
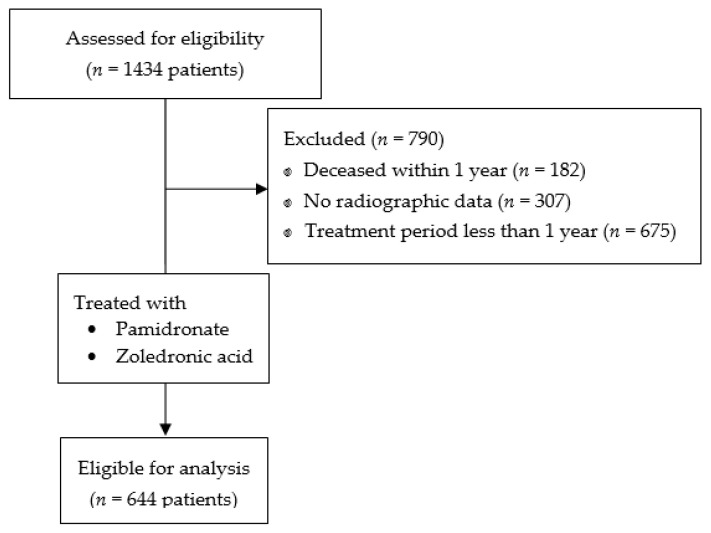
Flow diagram for the determination of the study population for assessment.

Extraction of data from the clinical data warehouse (CDW) of a single institute with a high-volume hematology hospital and a dental hospital, data of a total of 1434 patients who were diagnosed with multiple myeloma (ICD-10 C90, C900 with confirmed diagnosis) and treated with bisphosphonates (intravenous pamidronate or zoledronate) in periods January 2009 through December 2019 were obtained, then assessed for eligibility. Patients who deceased within one year of diagnosis, were treated with bisphosphonates for less than one year, had a previous history of treated osteoporosis, were ever treated with other class agents (e.g., denosumab), or had insufficient radiographic/clinical data were excluded from the study. Medical charts, including dental records, were retrospectively reviewed, then plain anteroposterior and lateral view femur radiographs with supplementary bone scan images were thoroughly reviewed by three orthopedic surgeons for 644 eligible patients who met the criteria (Figure 1).

The American Society for Bone and Mineral Research (ASBMR) task force 2013 revised definition of AFF [22] was used to evaluate the presence of AFFs or incomplete AFFs. The staging system suggested in the 2009, 2014 updated position paper from the American Association of Oral and Maxillofacial Surgeons [19,23] was used for the diagnosis of MRONJ in this study, when all patients were referred to the dental clinic for an oral health assessment with or prior to the initiation of the bisphosphonate therapy. Chance-corrected kappa coefficient of inter-observer variability for diagnosing AFF was 0.88. 

For independent variables, age, sex, body weight (kg), body mass index (BMI, kg/m^2^), total doses of bisphosphonate used, follow-up period, and treatment periods including a complete history of prescribed doses were collected. By reviewing the medical records, a considerable portion of the population had a prescription history of switching pamidronate or zoledronate to one another. Adjustments were necessary before arithmetically unifying the total dose to compare the accumulative dose of the two bisphosphonates into one variable. To adjust the relatively powerful potency of zoledronate over pamidronate, 100-times weight was applied to accumulative zoledronate doses in milligrams [24,25,26] for the addition with pamidronate dose. After the simple sum of total accumulative dose (mg) of the two bisphosphonates was calculated, these per BMI (kg/m^2^) and per body weight (kg) as well as potency-weighted accumulative dose (mg) per body weight (kg) were also assessed as variables for each complication. 

Incidences of AFF and MRONJ in the study population were initially calculated, then risk factor analyses were carried out to select a variable for subsequent cutoff titration to obtain the safety limit dose. Primary outcomes were separately recorded for surgical intervention on the femur as one endpoint and prominent radiologic evidence of impending or apparent AFF as another. Univariate logistic regression for all dependent variables, then multiple logistic regression were conducted to search for the risk factors and set a variable for subsequent analysis using the receiver operating characteristics (ROC) curve analysis to titrate cutoff values for the safety limit of accumulative dosages. The area under the curves (AUC) was again tested by Pearson chi-square to obtain odds ratios for each complication. *p*-values under 0.05 were considered statistically significant. All statistical analyses were conducted using SPSS (version 26.0, SPSS Inc., Chicago, IL, USA).

## 3. Results

Out of 644 patients eligible for analysis, the incidence of AFF in this study population was 0.93% (6/644) which underwent surgical intervention due to prominent fracture, while distinct radiographic abnormalities of impending AFF regardless of their treatment were found in 3.88% (25/644). MRONJ was found in 11.8% (76/644) of the population and concomitant presentation within a patient of both AFF and MRONJ was found in only 0.31% (2/644). MRONJ-diagnosed patients were treated with curettage and/or sequestrectomy without extraction (31.6%) under local anesthesia, with extraction (30.3%) under local anesthesia, or only conservative care including antibiotics therapy (22.4%), while some underwent surgical debridement and extraction under general endotracheal anesthesia (15.8%).

For demographics among the study population, 325 (50.5%) were men and 319 (49.5%) were women, with a mean follow-up period of 69.8 ± 33.1 (months, 12.1–128.3), mean age of 67.2 ± 9.5 (years, 18–89), mean body weight of 60.8 ± 13.1 (kg, 31.2–108), and mean BMI of 23.7 ± 3.6 (kg/m^2^, 13.5–62.2). For pharmacological options of bisphosphonate treatment, 381 (58.4%) were solely administrated with pamidronate, 18 (2.8%) exclusively used zoledronate, and 245 (30.9%) used a combination of pamidronate and zoledronate, where none of which were prescribed simultaneously (Table 1).

By univariate logistic regression, impending AFF as a dependent variable showed no significant results. However, for prominent AFFs which required surgery, accumulative zoledronate dose (OR = 1.017, *p* = 0.009), with that per BMI (OR = 1.013, *p* = 0.716), per weight (OR = 2.641, *p* = 0.009), and potency-weighted accumulative dose per body weight (OR = 1.010, *p* = 0.005) showed a significant increase in risks. Upon subsequent multiple logistic regression among significant variables, the potency-weighted sum of accumulative dose per body weight had significant results with the lowest *p*-values for both AFF (OR = 1.010, *p* = 0.005) and MRONJ (OR = 1.007, *p* < 0.001). In summary, logistic regression for risk factor analyses showed similar results for both AFF and MRONJ, except for the difference in results for the simple accumulative dose of zoledronate, which was significant for AFF (OR = 1.017, *p* = 0.009), but not for MRONJ (OR = 1.008, *p* = 0.675) (Table 2).

For the cutoff value analysis, the sum of potency-weighted accumulative dose (mg) per body weight (kg) was selected among the variables. ROC curve analysis was used to titrate the cutoff by selecting the value where the sum of sensitivity (Sn) and specificity (Sp) is maximized for AFF and MRONJ, respectively. As a result, for AFF, the cutoff value of 77.00 mg/kg (Sn = 0.833, Sp = 0.662, AUC = 0.762, *p* = 0.027) was titrated, and for MRONJ, 57.70 mg/kg was titrated (Sn = 0.712, Sp = 0.564, AUC = 0.661, *p* < 0.001). In turn, the Pearson chi-square test revealed elevated risks when exceeding the safety dose for AFF (OR = 7.300, *p* = 0.035) and MRONJ (OR = 3.201, *p* < 0.001) (Table 3).

## 4. Discussion

High-dose intravenous bisphosphonate treatment is inevitable in multiple myeloma patients due to its highly aggressive disease burden and poor survival (recent 5-year survival rate of about 50% [27,28]), despite its high risks of devastating skeletal complications of AFF and MRONJ. The incidence of prominent AFF was 0.93% (6/644) in the study population, which is approximately 37-times higher than the previously reported cumulative incidence of AFF after bisphosphonate treatment for typical osteoporosis treatment in the Korean cohort (0.025%, 13/10,333) [20]. The incidence of MRONJ was found to be 11.8% (76/644), which is about 118-times higher than the reported cumulative incidence from a larger Korean cohort (0.10%, 166/164,926) [21]. 

Incidences of skeletal complications were compared in terms of incidences from Korean cohorts to minimize the selection bias. For comparison of skeletal complications in anti-osteoporotic treatments, cumulative incidences of the cohorts were used because most of the reported incidences in the previous literatures were in terms of incidence rates or crude incidence rates. Incidence rate includes the concept of observed time, which is unsuitable for comparison in myeloma patients due to the discrepancies of different administration dosing and interval. 

Patients treated less than 12 months were excluded from the assessment because AFF and MRONJ can be considered as longer-term (>12 months) complications even for high-dose bisphosphonate administration. Prolonged use of frequent (e.g., monthly) bisphosphonates (>12 months) and oral trauma, including tooth extraction, are the main risk factors of ONJ [29]. Cumulative hazards for developing MRONJ were reported to be 0–1% by 12-months [30,31] use of high-dose bisphosphonates, which drastically elevated to 21% at three years [30] or 15% at four years of zoledronate treatment [30]. Studies on AFF risks in MM have been reported less [32,33]; AFF occurred as early as a cumulative dose of 24 bisphosphonates cycles, or 22 months in a similar study for breast cancer patients [34]. 

At the initial retrieval of study data using CDW, cases of treatment using bisphosphonates (e.g., alendronate) other than the standard treatment of pamidronate and zoledronate were excluded from the study. Denosumab (Xgeva^®^) has been known to have a considerable effect in preventing bone resorption by MM. However, it was also excluded because its use as a treatment for myeloma is gaining popularity only recently [35,36]. Current guidelines still include pamidronate or zoledronate only (clodronate used in exceptional cases) [9,37,38], hence we limited the study to pamidronate and zoledronate [37]. 

Overviewing data of prescription histories over the study period, pamidronate was mainly used in the past, but the recent trend is changing to favor zoledronate over pamidronate. Most of the cases using zoledronate were initially treated with short-to-long periods of pamidronate, the switched to zoledronate due to health insurance policies regarding zoledronate use for cancer patients in South Korea. This trend led us to deliberate on the necessity of formulation that can encompass both terms of accumulative dosages. Relative potency for inhibiting bone resorption of zoledronate over pamidronate has been reported in a wide range of discrepancies from 67 to 850-fold [25,39,40,41,42,43,44]. In this study, we set a 100-fold adjustment to zoledronate dose over pamidronate according to the American Society of Clinical Oncology (ASCO) practice guideline update in 2018 [9], for a reasonable comparison as well as for the use in arithmetic integration of the two bisphosphonate doses. 

Additionally, guidelines do not consider each patient’s body weight or BMI, as generally carried out in chemotherapies. Safety limits can be calculated easily by reorganized expressions (Table 3) using only the number of prescriptions of each bisphosphonate and the body weight of the patient. For example, for AFF, a 60 kg patient as a brief average of women in Europe, can bear 11.5 months and 51.3 months (4.3 years), respectively, before the odd ratio increases to 7.300 (*p* = 0.035), when intravenous zoledronate or pamidronate have been exclusively used as in the guideline [9]. An 80 kg patient as a brief average of men in Europe can bear 15.4 months and 68 months (5.7 years), respectively, for the same condition. For MRONJ, a 60 kg patient is relatively safe to administer for the duration of 8.6 months and 38.4 months (3.7 years), and an 80 kg patient is allowed for 11.5 months and 51.3 months (34.3 years), respectively, for intravenous zoledronate and pamidronate. Periods for the Asian population with relatively lower body weight would be shortened. Mixed use of the two agents can also be easily substituted for customized circumstances of each patient. However, the risk-benefits should be well considered, as utilizing weight-based dose adjustment to prevent relatively rare complications such as AFF and MRONJ might harm the benefits of bisphosphonates on MBD at the same time [45]. Reducing bisphosphonate duration would be detrimental to preventing major MBDs or extra-skeletal complications of myeloma.

In summary, re-evaluation of the patient and consultation with orthopedic surgery and dentistry to screen for skeletal complications are recommended in roughly one year of administration for zoledronate and four years for pamidronate. A periodic (e.g., every six months) dental examination with oral health education is required during the therapy [29]. More importantly, when tooth extraction is required, a drug holiday is essential to ensure optimal healing [29,46]. Additionally, routine serum creatinine level check-ups are required to monitor the effects of renal toxicity [9]. 

**Figure 2:**
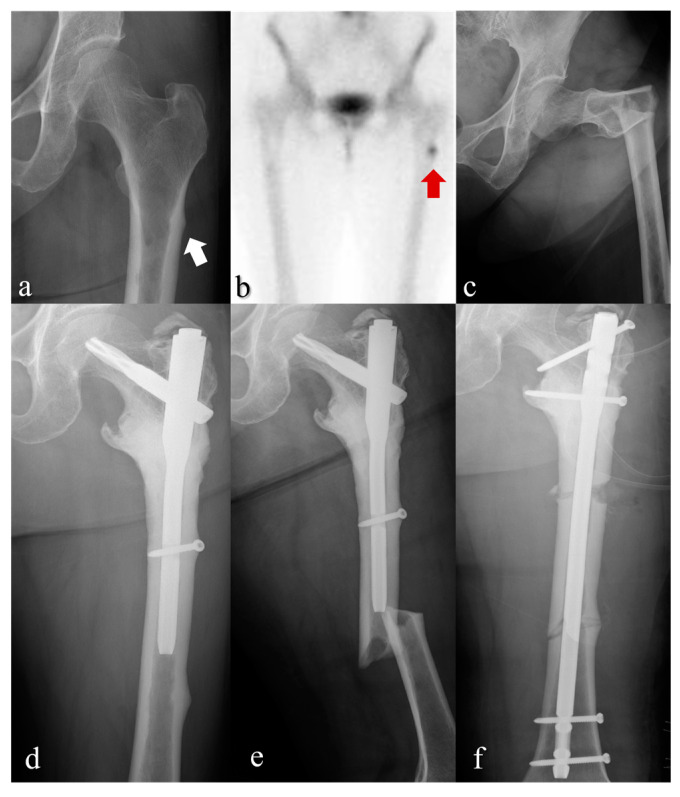
Anteroposterior view radiographs of the left femur (**a**,**c**–**f**) and a bone scan image (**b**) of two consecutive AFFs (arrows) occurred in a patient treated with high-dose bisphosphonates. (AFF: atypical femoral fracture).

We hereby present a case of a 67-year-old female patient with multiple myeloma referred from the hematology hospital suffering left hip pain after tripping. The patient was on high dose pamidronate (accumulative 540 mg for six months) before the first AFF occurred, where lateral cortex thickening on the hip radiograph (Figure 2a) and correlated hot uptake in the bone scan image was present (Figure 2b). At presentation, subtrochanteric AFF was diagnosed (Figure 2c) which was treated with intramedullary proximal femoral nail insertion (Figure 2d). After four years from the initial surgery, second AFF occurred more distally, where additional 144 mg of zoledronate (36 months period) had been administered to the patient between the two surgeries (Figure 2e). The patient was finally treated with full-length intramedullary femoral nail (Figure 2f).

There are a few limitations in this study. To overcome the pitfalls of a retrospective study, CDW was used to gain data from as many samples as possible. Exclusion of patients with short-term treatments (<12 months) would have overestimated the incidence to some extent, but we tried to focus on cumulative skeletal risks in the longer term. Additionally, the choice of agents in treating myeloma could have been skewed by domestic health insurance coverage standards, given that substitution of pamidronate with zoledronate during the treatment period were observed in a considerable proportion of the population. However, that is also why the cutoff value was determined using the sum of potency weighted accumulative dosage which incorporates both agents used in a patient. Lastly, the disease burden of myeloma itself was not considered in the study. The bone resorptive nature of myeloma burden itself could affect the occurrence of AFF or MRONJ, by encroaching on the cancellous and cortical structures of the bone. Future studies certainly must include the effects and safety of the promising agent, denosumab as well. 

## 5. Conclusions

The potency-weighted accumulative total dose of pamidronate and zoledronate (a 100-times) per body weight (kg) can provide a meaningful index for elevated risks in AFF and MRONJ when treating multiple myeloma patients with high-dose bisphosphonates. Body weight adjustments for accumulative dose calculation in terms of permissible dosing should be taken into consideration. When the accumulative treatment dose exceeds the cutoff of 77.00 mg/kg or 57.70 mg/kg, respectively, for AFF and MRONJ, or a treatment period over roughly one year for zoledronate or four years for pamidronate, radiologic evaluation of femurs, dental examination and a thorough re-evaluation on patient symptoms should be examined. Physicians might also consider a drug holiday or switching the treatment to a different class agent (e.g., denosumab). 

## Figures and Tables

**Table 1:** Demographics of 644 patients eligible for analysis.

Items	
Patients for assessment	644
Sex	
Male	325 (50.5%)
Female	319 (49.5%)
Mean follow-up ± SD (range), months	69.8 ± 33.1 (12.1–128.3)
Mean age ± SD (range), years	67.2 ± 9.5 (18–89)
Mean body weight ± SD (range), kg	60.8 ± 13.1 (31.2–108)
Mean BMI ± SD (range), kg/m^2^	23.7 ± 3.6 (13.5–62.2)
Bisphosphonate treatment options	
Pamidronate only	381 (59.2%)
Zoledronic acid only	18 (2.8%)
Pamidronate + zoledronic acid	245 (38.0%)

**Table 2:** Univariate and multivariate logistic regression analyses for risk factors of skeletal complications.

	Atypical Femoral Fracture (AFF)			Medication-Related Osteonecrosis of the Jaw (MRONJ)		
Item	OR	95% C.I.	*p*	OR	95% C.I.	*p*
Age	1.024	0.937–1.120	0.594	1.012	0.981–1.045	0.444
Sex (women: men)	2.063	0.375–11.349	0.405	0.511	0.245–1.064	0.073
Body weight (kg)	0.990	0.919–1.066	0.792	0.982	0.922–1.046	0.580
BMI (kg/m^2^)	0.983	0.778–1.241	0.885	1.014	0.865–1.189	0.863
Accumulative treatment dose (mg)						
Pamidronate	1.000	1.000–1.001	0.755	1.000	0.999–1.001	0.786
Zoledronate	1.017	1.004–1.029	0.009 ^†^	1.008	0.972–1.044	0.675
Accumulative dose(mg)/BMI (kg/m^2^)						
Pamidronate	1.013	0.947–1.083	0.716	1.002	0.998–1.006	0.351
Zoledronate	1.441	1.085–1.914	0.012 ^†^	1.262	1.110–1.435	<0.001 ^†^
Accumulative dose(mg)/Bwt (kg)						
Pamidronate	1.005	0.976–1.034	0.762	1.004	0.994–1.014	0.401
Zoledronate	2.641	1.277–5.462	0.009 ^†^	1.816	1.313–2.513	<0.001 ^†^
Potency-weighted accumulative dose (mg)/Bwt (kg)						
Pamidronate	1.010	0.976–1.034	0.762	1.004	0.994–1.014	0.401
Zoledronate	1.010	1.002–1.017	0.009 ^†^	1.006	1.003–1.009	<0.001 ^†^
Pamidronate + zoledronate total dose	1.010	1.003–1.017	0.005 ^†^	1.007	1.003–1.010	<0.001 ^†^

^†^ These *p*-values were less than 0.05. OR indicates odds ratio; C.I. for confidence interval; Bwt for body weight.

**Table 3:** Suggested cutoff for increased risks for AFF (OR = 7.300, *p* = 0.035) and MRONJ (OR = 3.201, *p* < 0.001).

$\frac{Potency-weighted\;accumulative\;dose\;\left(mg\right)}{\mathrm{Body}\;\mathrm{weight}\;\left(kg\right)}$ $=\{\begin{array}{l} 77.00\;\left(AFF\right)\\ 57.70\;\left(MRONJ\right) \end{array}$
⸫ for AFF: pamidronate ∗ 90 mg + zoledronate ∗ 4 mg ∗ 100 ≥ 77 ∗ Bwt (kg)
⸫ for MRONJ: pamidronate ∗ 90 mg + zoledronate ∗ 4 mg ∗ 100 ≥ 57.70 ∗ Bwt (kg)

## Data Availability

The datasets used and analyses are available from the corresponding author upon reasonable request.
